# Outcomes after a second allogeneic haematopoietic stem cell transplant for relapsed paediatric acute myeloid leukaemia improved over time: A study from the EBMT Paediatric Diseases Working Party

**DOI:** 10.1111/bjh.70167

**Published:** 2025-09-30

**Authors:** Nimrod Buchbinder, Victor Michel, Arnaud Dalissier, Katharina Kleinschmidt, Franco Locatelli, Alexei Maschan, Robert Wynn, Franca Fagioli, Marco Zecca, Charlotte Jubert, Birgitta Versluys, Petr Sedlacek, Ludmila Zubarovskaya, Marta Gonzalez Vicent, Alessandra Biffi, Gérard Michel, Oana Mirci‐Danicar, Wolfgang Holter, Marc Ansari, Jacques‐Emmanuel Galimard, Pascale Schneider, Mouad Abouqateb, Krzysztof Kałwak

**Affiliations:** ^1^ Service d'Immuno‐Hémato‐Oncologie Pédiatrique Hôpital Charles Nicolle Rouen France; ^2^ EBMT Paris Study Unit Saint Antoine Hospital, INSERM UMR‐S 938, Sorbonne University Paris France; ^3^ Department of Pediatric Hematology and Oncology IRRCS Ospedale Pediatrico Bambino Gesù Catholic University of the Sacred Heart Rome Italy; ^4^ Federal Research Center for Pediatric Hematology Moscow Russian Federation; ^5^ Department of Paediatric Haematology, Bone Marrow Unit Royal Manchester Children's Hospital Manchester UK; ^6^ Onco‐Ematologia Pediatrica, Centro Trapianti Cellule Staminali Ospedale Infantile Regina Margherita Torino Italy; ^7^ Fondazione IRCCS Policlinico San Matteo Pediatric Hematology‐Oncology Pavia Italy; ^8^ CHU Bordeaux Groupe Hospitalier Pellegrin‐Enfants Bordeaux France; ^9^ Princess Maxima Center/University Hospital for Children (WKZ), Stem Cell Transplantation Utrecht The Netherlands; ^10^ Department of Paediatric Haematology and Oncology University Hospital Motol Prague Czech Republic; ^11^ RM Gorbacheva Research Institute, Pavlov University Saint Petersburg Russian Federation; ^12^ Niño Jesus Children's Hospital Stem Cell Transplant Unit Madrid Spain; ^13^ Clinica di Oncoematologia Pediatrica Dipartimento di Pediatria Padova Italy; ^14^ Département Hématologie Oncologie Pédiatrique Hôpital de la Timone Marseille France; ^15^ Department of Paediatric Oncology/BMT Bristol Royal Hospital for Children Bristol UK; ^16^ Stem Cell Transplantation Unit St. Anna Kinderspital Vienna Austria; ^17^ Cansearch Research Platform for Pediatric Oncology and Hematology, Faculty of Medicine, Department of Pediatrics, Gynecology and Obstetrics University of Geneva Geneva Switzerland; ^18^ Division of Pediatric Oncology and Hematology, Department of Women, Child and Adolescent University Geneva Hospitals Geneva Switzerland; ^19^ Department of Pediatric Hematology/Oncology BMT Wroclaw Medical University Wroclaw Poland

**Keywords:** acute leukaemia, paediatric haematology, stem cell transplantation

## Abstract

Evolution of acute myeloid leukaemia (AML) treatments and transplantation procedures may affect outcomes after second haematopoietic stem cell transplantation (HSCT2) for relapsed paediatric AML. We analysed 345 paediatric patients reported to the European Society for Bone Marrow Transplantation (EBMT) registry for HSCT2 performed for AML relapse post‐HSCT between 2000 and 2022. Multivariable analyses were adjusted for sex, age, transplant period, donor, disease status pre‐HSCT2, cytogenetics, conditioning, total body irradiation (TBI) and post‐first haematopoietic stem cell transplantation (HSCT1) remission duration. At three years leukaemia‐free survival (LFS), overall survival (OS), non‐relapse mortality (NRM), relapse incidence (RI) and graft‐versus‐host disease (GVHD)/relapse‐free survival (GRFS) were 30.2%, 37.5%, 19.1%, 50.7% and 20.7% respectively. Compared with the 2000–2013 period, HSCT2 performed in 2014–2022 had better LFS (hazard ration [HR]: 0.66, 95% confidence interval [95% CI]: 0.48–0.90; 3‐year: 34.3% vs. 26.3%), OS (HR: 0.60, 95% CI: 0.42–0.84; 3‐year: 42.9% vs. 32.8%), RI (HR: 0.66, 95% CI: 0.46–0.98; 3‐year: 46.0% vs. 54.7%) and GRFS (HR: 0.65, 95% CI: 0.48–0.90; 3‐year: 25.3% vs. 16.1%) while NRM and GVHD incidence were stable. Relapse >6 months post‐HSCT1 and remission pre‐HSCT2 were associated with better LFS, OS and RI. Conditioning and cytogenetics did not influence outcomes. Mismatched unrelated donor negatively affected OS. These results highlight the improving survival after HSCT2 and support it in selected patients, particularly those relapsing later and in remission at HSCT2.

## Graphical abstract

We retrospectively evaluated outcomes after a second haematopoietic stem cell transplantation (HSCT) performed between 2000 and 2022 for relapsed acute myeloid leukaemia in 345 children. We found that leukaemia‐free survival, overall survival, graft‐versus‐host disease (GVHD)/relapse‐free survival (GRFS) and relapse incidence improved over time, while toxicity related mortality (TRM) and GVHD rates remained stable. Late relapses occurring more than 6 months and complete remission achieved before the second transplant were the two main predictors of a better outcome. A mismatched related donor (mostly haplo) was associated with poorer overall survival. No impact was found for cytogenetic subgroup or conditioning procedure.

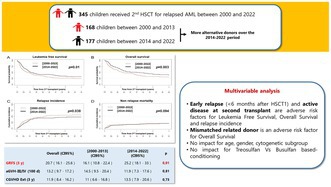

## INTRODUCTION

Significant progress has been achieved in treating paediatric acute myeloid leukaemia (AML) over the past 50 years, with overall survival (OS) rates now reaching 70%–75%.^1^, ^2^, ^3^, ^4^ A large part of these improvements lies in more effective first‐line therapeutic strategies, including refined cytogenetic and molecular stratification, minimal residual disease monitoring, haematopoietic stem cell transplantation (HSCT) in first complete remission (CR1) and development of new treatment options for relapsed/refractory disease. At the same time, the enhanced supportive care has led to a reduction in treatment‐related toxicities, which contribute to paediatric AML outcomes.^5^, ^6^, ^7^, ^8^, ^9^, ^10^, ^11^

It is worth noting that HSCT remains the only possible cure in 40% of cases, including relapsed and refractory diseases often associated with intermediate/high cytogenetic/molecular risk features.^6^, ^12^ Several pre‐emptive and prophylactic strategies involving immunomodulation, targeted treatments (i.e.  FLT3 inhibitors) or hypomethylating agents are commonly used to prevent relapse after HSCT.^13^ However, the effectiveness of these treatments in preventing post‐transplant relapse has not been fully established, particularly in the paediatric setting. Thus, although HSCT provides these children with an opportunity for long‐term survival, we still encounter a high relapse rate of approximately 30%, with a median survival time of only 5 months for these patients.^10^, ^14^, ^15^, ^16^

In case of post‐transplant relapse, a second allogeneic haematopoietic stem cell transplantation (HSCT2) may be a curative option but comes with a high risk of both new relapse and severe toxicity in this heavily treated population. Besides small series, two extensive retrospective studies have primarily documented the outcomes following HSCT2 for post‐transplant paediatric relapsed AML.^14^, ^17^, ^18^, ^19^, ^20^, ^21^, ^22^

In 2018, Yaniv et al. conducted a retrospective study based on 373 patients registered in the European Society for Bone Marrow Transplantation (EBMT) database who underwent HSCT2 for post‐transplant acute leukaemia (either lymphoblastic or myeloblastic) relapse between 2004 and 2013. In the AML group (*n* = 159), 5‐year OS and leukaemia‐free survival (LFS) probabilities were 24% and 17% respectively.^23^ The second retrospective study, published by Uden et al. in 2020, presented the outcomes of 333 patients treated in I‐BFM (international Berln‐Frankfurt‐Munchen) centres for post‐transplant relapse. Among these patients, 122 received HSCT, with a 4‐year OS probability of 31%.^24^ Both studies identified relapse as the primary reason for failure following HSCT2. The 4‐year cumulative incidence of relapse (RI) was 45% after HSCT2 in the I‐BFM cohort, while Yaniv reported a 5‐year RI of 65%. Non‐relapse mortality (NRM) was also significant, reaching 22% in both cohorts.^23^, ^24^

In recent years, several advances in both AML and allograft management may have improved the prognosis of second allografts for AML. These advances include the development of new therapeutic tools for refractory/relapsed AML, like venetoclax or CPX‐351, broader use of potentially less toxic treosulfan‐based myeloablative conditioning (MAC) regimens, improved management of alternative donors' transplantation and the implementation of different pre‐emptive and prophylactic post‐transplant strategies.^25^, ^26^, ^27^, ^28^, ^29^

In this study, we conducted a retrospective registry‐based analysis among a large international cohort of children receiving HSCT2 for AML relapse after HSCT1. We aimed to analyse trends in patient characteristics, transplant settings and outcomes over the last two decades and identify predictive factors that might allow us to identify the best children to benefit from this procedure.

## METHODS

### Study design

We conducted a retrospective, multicentre study and analysis. The Paediatric Diseases Working Party (PDWP) of the EBMT institutional review board approved the study. Data were provided by the EBMT PROMISE database. All patients and/or guardians provided written informed consent for data entry into the ProMISe database and its use for analysis, per the Declaration of Helsinki. The study included patients under 18 years who received a second allogeneic HSCT between 2000 and 2022, with a diagnosis of AML at the first and second transplantation. The information was gathered from the EBMT ProMISe database and comprised demographics, diagnosis, disease status, graft characteristics, conditioning regimen and graft‐versus‐host disease (GVHD) prophylaxis for both transplants.

### Definitions

Responses to treatment were classified in accordance with the 2022 European LeukemiaNet (ELN) criteria. Relapse after HSCT1 was defined as more than 5% bone marrow blasts or reappearance of circulating blasts. The cytogenetic risk subgroups were assessed according to the 2022 ELN criteria. Because of missing molecular data, only the cytogenetic component of risk classification was used for this purpose. MAC was defined using EBMT guidelines. Neutrophil polymorphonuclear (PMN) cell engraftment was defined as absolute neutrophil count (ANC) ≥ 0.5 × 10^9/L^ (≥500/μL) for three consecutive days. Platelet engraftment was defined as absolute platelet count ≥20 × 10^9/L^ (≥20 000/μL), without transfusion support for seven consecutive days.

### Statistics

Median values, interquartile ranges (IQRs), and minimum and maximum values were used to describe quantitative variables; frequency and percentage were used for categorical variables. Primary patient‐, disease‐, donor‐ and transplant‐related characteristics were compared using Pearson's chi‐squared test for categorical variables and the Mann–Whitney U test for quantitative variables.

Study endpoints were LFS, OS, NRM, RI, GVHD‐free/relapse‐free survival (GRFS) and cumulative incidence of acute GVHD (aGVHD) and chronic GVHD (cGVHD). These endpoints were determined for the whole population and for two different periods: 2000–2013 and 2014–2022. This cut‐off was determined because the population median was located in 2014. The initial time was the date of the second HSCT for all endpoints. LFS was defined as survival with no evidence of relapse or progression. OS was defined as time to death from any cause. NRM was defined as death from any cause without previous relapse or progression. GRFS was defined as survival without incidence of relapse, grade III–IV aGVHD or extensive cGVHD. Probabilities of OS, PFS and GRFS were calculated using the Kaplan–Meier method. Cumulative incidence was used to estimate NRM, RI, as well as acute and chronic GVHD in a competing risk setting, where death and relapse were considered as competing risks as appropriate. Multivariable analyses were performed using the Cox cause‐specific proportional‐hazards model for all endpoints. In the primary analysis, the models included the period of transplant, type of donor, age, recipient gender and disease stage at HSCT2, cytogenetics, conditioning, use of total body irradiation (TBI) for HSCT2 and the interval between HSCT1 and relapse. Additionally, interaction terms between the period and adjustment variables were assessed for our primary outcome and for outcomes where the period is significant. For the secondary analysis comparing busulfan (Bu) versus treosulfan (Treo) conditioning regimens, we used a backward stepwise selection based on the Akaike information criterion (AIC) to identify key adjustment factors; these included age at transplant, the delay between HSCT1 and relapse, and the year of transplant as the key statistical adjustment factors, and given its clinical significance, disease status at transplant was also included in the final models. The centre effect was considered by introducing a random or frailty effect into all models. Results were expressed as the hazard ratio (HR) with a 95% confidence interval (95% CI). All tests were two‐sided with a type I error rate fixed at 0.05.

Statistical analyses were performed with R 4.3.0 software (R Development Core Team, Vienna, Austria) packages.

## RESULTS

### Patient and transplant characteristics

A total of 345 patients received HSCT2 for AML relapse from 2000 to 2022. The median age at HSCT2 was 10.4 years (Q1: 5.2; Q3: 14.5). The median time between HSCT1 and relapse was 10.8 months (Q1: 6.2; Q3: 17.8). Most AML relapses displayed intermediate‐risk cytogenetics (*n* = 127, 55.2%). Concerning disease status at HSCT2, 265 patients (76.8%) were in CR, mainly second CR (*n* = 175, 50.7%).

A different donor was chosen for 73.7% of HSCT2. Donors were unrelated (UD) in 52.6% (*n* = 181), matched related (MRD) in 22.4% (*n* = 77) and mismatched related (MMRD) in 25% (*n* = 86). Most MMRD were haploidentical (20.6% of the total patient population). Peripheral blood (PB) was the most common stem cell source (*n* = 175, 50.9%), followed by bone marrow (BM) (*n* = 112, 32.6%) and cord blood (CB) (*n* = 57, 16.6%). In vivo T‐cell depletion was performed in 51.6%, primarily with anti‐thymocytes globulins (ATG). Ex vivo T‐cell depletion was used in 54 transplants (16.2%). Pretransplant cytomegalovirus (CMV) seropositivity in donors and recipients was 49.3% and 64.3% respectively. Donor/recipient‐specific positivity/negativity is shown in Table 1.

**TABLE 1:** Patients and second transplant characteristics and evolution over time.

	Total (*N* = 345)	[2000–2013] (*N* = 168)	[2014–2022] (*N* = 177)	*p*‐value
Age at transplant, yrs				0.73
Median [Q1, Q3]	10.4 (5.2, 14.5)	10.4 (5.3, 14.6)	10.1 (4.7, 14.5)	
[Min, Max]	0.9–18.0	0.9–18.0	0.9–17.9	
Age at diagnosis, yrs				0.43
Median [Q1, Q3]	7.4 (2.7, 12.0)	7.5 (2.8, 12.3)	7.0 (2.5, 11.6)	
[Min, Max]	0.0–17.1	0.0–17.0	0.1–17.1	
Missing count	2	1	1	
Patient sex at birth				0.96
Male	209 (60.6%)	102 (60.7%)	107 (60.5%)	
Female	136 (39.4%)	66 (39.3%)	70 (39.5%)	
Lansky score				0.06
<90	73 (29.4%)	36 (36.0%)	37 (25.0%)	
≥90	175 (70.6%)	64 (64.0%)	111 (75.0%)	
Missing count	97	68	29	
AML cytogenetics				0.63
Adverse	80 (34.8%)	28 (35.0%)	52 (34.7%)	
Favourable	23 (10.0%)	10 (12.5%)	13 (8.7%)	
Intermediate	127 (55.2%)	42 (52.5%)	85 (56.7%)	
Missing count	115	88	27	
Delay first allo to relapse, months				0.05
Median [Q1, Q3]	10.8 (6.2, 17.8)	10.1 (5.7, 17.0)	11.6 (6.5, 20.3)	
[Min, Max]	0.1–74.2	0.6–56.8	0.1–74.2	
Missing count	34	17	17	
Type of donor (regrouped)				<0.01
Matched_related_donor	77 (22.4%)	54 (32.3%)	23 (13.0%)	
Mismatch_related_donor	86 (25.0%)	34 (20.4%)	52 (29.4%)	
Unrelated_Donor	181 (52.6%)	79 (47.3%)	102 (57.6%)	
Missing count	1	1	0	
Type of donor				
Haplo	71 (20.6%)	21 (12.6%)	50 (28.2%)	
MMR (missing HLA)	12 (3.5%)	11 (6.6%)	1 (0.6%)	
MMR 1 locus	3 (0.9%)	2 (1.2%)	1 (0.6%)	
MOR	6 (1.7%)	3 (1.8%)	3 (1.7%)	
MSD	71 (20.6%)	51 (30.5%)	20 (11.3%)	
UCB	57 (16.6%)	11 (6.6%)	46 (26.0%)	
UD (missing HLA)	43 (12.5%)	29 (17.4%)	14 (7.9%)	
UD ≤ 8/10	2 (0.6%)	1 (0.6%)	1 (0.6%)	
UD 10/10	61 (17.7%)	26 (15.6%)	35 (19.8%)	
UD 9/10	18 (5.2%)	12 (7.2%)	6 (3.4%)	
Missing count	1	1	0	
Donor to patient CMV positivity				0.01
Neg to Neg	76 (26.6%)	39 (29.5%)	37 (24.0%)	
Neg to Pos	69 (24.1%)	37 (28.0%)	32 (20.8%)	
Pos to Neg	26 (9.1%)	16 (12.1%)	10 (6.5%)	
Pos to Pos	115 (40.2%)	40 (30.3%)	75 (48.7%)	
Missing count	59	36	23	
Same donor as previous HSCT				<0.01
No	196 (73.7%)	62 (56.9%)	134 (85.4%)	
Yes	70 (26.3%)	47 (43.1%)	23 (14.6%)	
Missing count	79	59	20	
Disease status at HSCT2				0.99
Complete remission	265 (76.8%)	129 (76.8%)	136 (76.8%)	
Relapse	80 (23.2%)	39 (23.2%)	41 (23.2%)	
Disease status at HSCT2				0.71
Rel1	55 (15.9%)	26 (15.5%)	29 (16.4%)	
CR2	175 (50.7%)	85 (50.6%)	90 (50.8%)	
Rel2	23 (6.7%)	11 (6.5%)	12 (6.8%)	
CR ≥ 3	90 (26.1%)	44 (26.2%)	46 (26.0%)	
Rel ≥ 3	2 (0.6%)	2 (1.2%)	0 (0.0%)	
MRD				0.15
Active disease	80 (44.2%)	39 (51.3%)	41 (39.0%)	
MRD neg	77 (42.5%)	26 (34.2%)	51 (48.6%)	
MRD pos	24 (13.3%)	11 (14.5%)	13 (12.4%)	
Missing count	164	92	72	
TBI				0.38
No	227 (67.4%)	108 (65.1%)	119 (69.6%)	
Yes	110 (32.6%)	58 (34.9%)	52 (30.4%)	
Missing count	8	2	6	
Myeloablative conditioning				<0.01
No	89 (26.7%)	57 (35.8%)	32 (18.4%)	
Yes	244 (73.3%)	102 (64.2%)	142 (81.6%)	
Missing count	12	9	3	

Abbreviations: AML, acute myeloid leukaemia; CMV, cytomegalovirus; CR, complete remission; Haplo, haploidentical; HSCT, allogeneic stem cell transplantation; HSCT1, first HSCT; HSCT2, second HSCT; MMR, mismatched related; MOR, matched other related; MRD, minimal residual disease; MSD, matched sibling donor; Neg, negative; Pos, positive; Rel, relapse; TBI, total body irradiation; UCB, unit cord blood; UD, unrelated donor; Yrs, years.

MAC regimen was used in 75.2% of HSCT2. Among different procedures, we found total body irradiation in 34.4%, busulfan in 23.1% and treosulfan in 23.8% of transplants. Other drugs included thiotepa, cyclophosphamide, fludarabine and melphalan. GVHD prophylaxis was predominantly ciclosporin based (*n* = 219, 76.3%).

One hundred and sixty‐eight patients were transplanted between 2000 and 2013, and 177 between 2014 and 2022. The median time from HSCT1 to relapse was 10.1 months in the earlier period, and 11.6 months in the more recent period (*p =* 0.05). The time between relapse and HSCT2 was 3.2 months in the earlier period and 3.4 months in the recent period (*p =* 0.05). The overall time interval between the HSCT1 and HSCT2 increased from 13.8 to 16.7 months (*p =* 0.01). There was no significant difference in disease status at transplant between the two periods, with CR achieved before HSCT2 in 76.8% of patients for both groups (*p =* 0.71) nor in performance status (2000–2013: LS ≥90% in 64% of patients, 2014–2022: increase to 75%, *p =* 0.06) at the time of HSCT2.

Evolution in transplant settings between the two periods was noted mainly for donor type (more haploidentical donors in the recent period), stem cells source (more CB units and PB stem cells in the recent period), and conditioning regimen (more often myeloablative, increase in both busulfan‐ and treosulfan‐based conditioning). All the differences between those two chronological groups' baseline characteristics are presented in Table 1 and Table S1.

### Outcomes

The median follow‐up after HSCT2 was 4.9 years (95% CI: 4.4–6) in the overall cohort, 10 years (95% CI: 8.4–10.9) in patients transplanted between 2000 and 2013, and 3 years (95% CI: 1.9–4) in the most recent period. The cumulative incidences of PMN and platelet engraftment were 89.2% (95% CI: 85.1–92.2) and 82.6% (95% CI: 76.8–87), respectively, and were similar over time (Table 2).

**TABLE 2:** Outcomes after second allogeneic stem cell transplantation and evolution over time.

	Overall (95% CI)	[2000–2013] (95% CI)	[2014–2022] (95% CI)
Median FU (y)	4.9 (4.4–6)	10 (8.4–10.9)	3 (1.9–4)
3‐y LFS (%)	30.2 (24.9–35.5)	26.3 (19.7–33.4)	34.3 (26.2–42.5)
3‐y NRM (3%)	19.1 (14.9–23.7)	19 (13.4–25.4)	19.7 (13.5–26.8)
3‐y RI (3%)	50.7 (44.9–56.2)	54.7 (46.6–62)	46 (37.6–54)
3‐y OS (%)	37.5 (31.8–43.2)	32.8 (25.6–40.2)	42.9 (34–51.6)
3‐y GRFS (%)	20.7 (16.1–25.6)	16.1 (10.8–22.4)	25.2 (18.1–33)
D + 100 aGVHD II–IV (%)	34.8 (29.5–40.1)	35.9 (28.5–43.3)	33.6 (26.2–41.2)
D + 100 aGVHD III–IV (%)	13.2 (9.7–17.2)	14.5 (9.5–20.4)	11.9 (7.3–17.6)
3‐y cGVHD (%)	20.8 (16.3–25.8)	20.6 (14.4–27.6)	20.8 (14.4–28)

Abbreviations: a/c GVHD, acute/chronic graft‐versus‐host disease; CI, confidence interval; d, day; FU, follow‐up; GRFS, graft‐versus‐host disease/relapse‐free survival; HR, hazard ratio; LFS, leukaemia‐free survival; NRM, non‐relapse mortality; OS, overall survival; *p*, *p*‐value; RI, relapse incidence; y, years.

**FIGURE 1:**
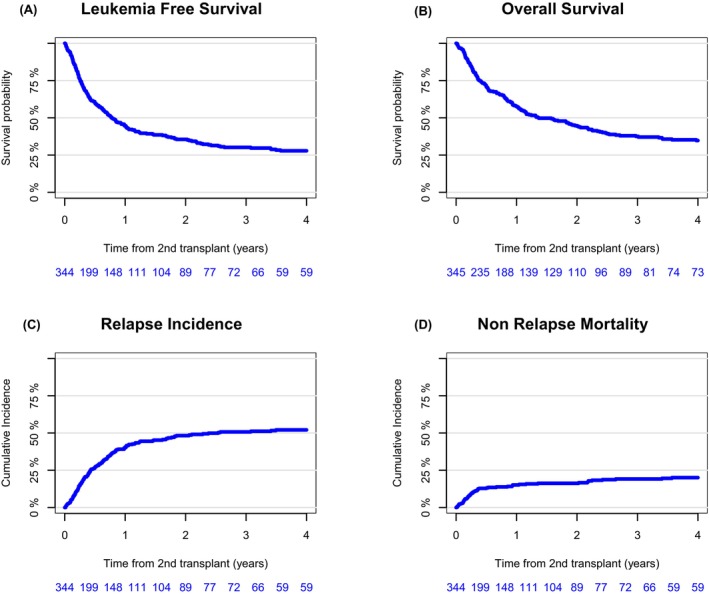
Outcomes after second allogeneic stem cell transplantation. Leukaemia‐free survival (A), overall survival (B), the cumulative incidence of relapse (C) and non‐relapse mortality (D) after second allogeneic stem cell transplantation in children with post‐transplant relapsed AML.

At three years LFS and OS were 30.2% (95% CI: 24.9–35.5) and 37.5% (95% CI: 31.8–43.2) respectively. Across the cohort, the rate of NRM at 3 years was 19.1% (95% CI: 14.9–23.7). The RI at 3 years was 50.7% (95% CI: 44.9–56.2), and GRFS was 20.7% (95% CI: 16.1–25.6). The incidence of grade II–IV aGVHD was 34.8% (95% CI: 29.5–40.1) and of grade III–IV 13.2% (95% CI: 9.7–17.2). At three years extensive cGVHD was 11.9% (95% CI: 8.4–16.2) (Figure 1; Table 2).

### Effect of HSCT2 period on patient outcomes

In the multivariable analysis, undergoing HSCT2 in the recent period (2014–2022) compared to the earlier period (2000–2013) was significantly associated with improved LFS (HR: 0.66 [95% CI: 0.48–0.90], *p* = 0.01; 3‐year probability: 34.3% vs. 26.3%), OS (HR: 0.59 [95% CI: 0.42–0.84], *p* = 0.003; 3‐year probability: 42.9% vs. 32.8%) and GRFS (HR: 0.66 [95% CI: 0.48–0.90], *p* = 0.01; 3‐year probability: 25.2% vs. 16.1%).

**FIGURE 2:**
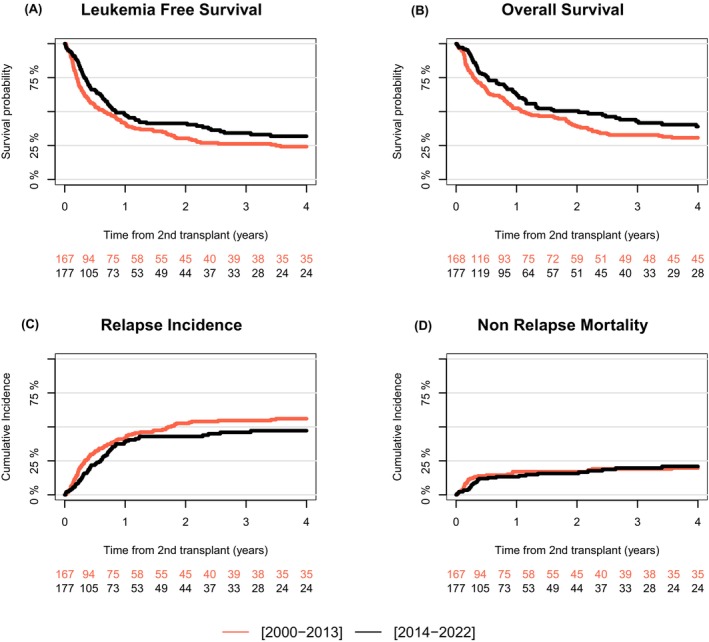
Outcomes over time after second allogeneic stem cell transplantation. Leukaemia‐free survival (A), overall survival (B), the cumulative incidence of relapse (C) and non‐relapse mortality (D) after second allogeneic stem cell transplantation for patients transplanted between 2000 and 2013 and 2014 and 2022.

These outcomes improvement between the two periods showed up with a markedly lower RI (HR: 0.67 [95% CI: 0.46–0.98], *p* = 0.038; 3‐year incidence: 46% vs. 54.7%) but no significant difference for NRM (HR: 0.58 [95% CI: 0.30–1.10], *p* = 0.094; 3‐year incidence: 19.7% vs. 19%) and GVHD‐related outcomes: aGVHD II–IV (HR: 0.89 [95% CI: 0.57–1.41], *p* = 0.63; 100‐day incidence: 33.6% vs. 35.9%), aGVHD III–IV (HR: 0.92 [95% CI: 0.46–1.84], *p =* 0.81; 100‐day incidence: 11.9% vs. 14.5%) and cGVHD (HR: 0.88 [95% CI: 0.43–1.80], *p =* 0.73; 3‐year incidence: 20.8% vs. 20.6%) (Figure 2; Tables 2 and 3; Table S2).

**TABLE 3:** Multivariable analysis of risk factors for outcomes after second allogeneic stem cell transplantation.

Characteristic	Leukaemia‐free survival (LFS)		Overall survival (OS)		Non‐relapse mortality (NRM)		Relapse incidence (RI)		GRFS	
	HR (95% CI)	*p*‐value	HR (95% CI)	*p*‐value	HR (95% CI)	*p*‐value	HR (95% CI)	*p*‐value	HR (95% CI)	*p*‐value
HSCT2 time period										
[2000–2013]	—		—		—		—		—	
[2014–2022]	0.66 (0.48 to 0.90)	**0.010**	0.59 (0.42 to 0.84)	**0.003**	0.58 (0.30 to 1.10)	0.094	0.67 (0.46 to 0.98)	**0.038**	0.66 (0.48 to 0.90)	**0.010**
Age at transplant^a^	1.02 (0.99 to 1.05)	0.29	1.02 (0.99 to 1.05)	0.16	1.05 (0.99 to 1.11)	0.094	1.01 (0.97 to 1.04)	0.67	1.02 (0.99 to 1.05)	0.29
Patient sex^a^										
Male	—		—		—		—		—	
Female	0.93 (0.69 to 1.26)	0.66	0.97 (0.70 to 1.35)	0.87	0.89 (0.48 to 1.65)	0.71	0.93 (0.65 to 1.33)	0.69	0.93 (0.69 to 1.26)	0.66
Delay HSCT1‐relapse, months										
0 to 6	—		—		—		—		—	
More than 6	0.60 (0.43 to 0.84)	**0.003**	0.64 (0.44 to 0.93)	**0.018**	0.61 (0.30 to 1.21)	0.16	0.59 (0.40 to 0.87)	**0.008**	0.60 (0.43 to 0.84)	**0.003**
Donor type										
Matched related Donor	—		—		—		—		—	
Mismatched related donor	1.52 (0.97 to 2.38)	0.068	1.65 (1.01 to 2.69)	**0.044**	1.50 (0.53 to 4.28)	0.44	1.52 (0.92 to 2.53)	0.10	1.52 (0.97 to 2.38)	0.068
Unrelated_Donor	1.22 (0.82 to 1.81)	0.33	1.14 (0.74 to 1.76)	0.55	1.83 (0.75 to 4.45)	0.18	1.07 (0.68 to 1.67)	0.78	1.22 (0.82 to 1.81)	0.33
Cytogenetic risk^a^										
Favourable & Intermediate	—		—		—		—		—	
Adverse	1.22 (0.85 to 1.76)	0.27	1.48 (1.00 to 2.20)	0.051	1.37 (0.70 to 2.68)	0.36	1.17 (0.76 to 1.81)	0.48	1.22 (0.85 to 1.76)	0.27
Missing	1.03 (0.72 to 1.47)	0.88	1.17 (0.79 to 1.74)	0.43	0.68 (0.31 to 1.48)	0.33	1.17 (0.78 to 1.77)	0.45	1.03 (0.72 to 1.47)	0.88
Disease status at transplant										
CR	—		—		—		—		—	
Active disease	1.75 (1.23 to 2.50)	**0.002**	1.72 (1.17 to 2.52)	**0.005**	1.45 (0.66 to 3.18)	0.35	1.87 (1.24 to 2.81)	**0.003**	1.75 (1.23 to 2.50)	**0.002**
TBI regimen										
No	—		—		—		—		—	
Yes	0.83 (0.59 to 1.16)	0.27	0.72 (0.49 to 1.04)	0.078	0.68 (0.34 to 1.37)	0.28	0.88 (0.60 to 1.30)	0.53	0.83 (0.59 to 1.16)	0.27
MAC regimen										
No	—		—		—		—		—	
Yes	1.00 (0.72 to 1.38)	0.99	1.16 (0.81 to 1.67)	0.43	1.30 (0.64 to 2.63)	0.46	0.94 (0.65 to 1.37)	0.75	1.00 (0.72 to 1.38)	0.99

*Note*: Significant values are in bold.

Abbreviations: CI, confidence interval; CR, complete remission; HR, hazard ratio; HSCT, allogeneic stem cell transplantation; HSCT1, first HSCT; HSCT2, second HSCT; MAC, myeloablative conditioning regimen; MMRD, mismatched related donor; MRD, matched related donor; Rel, relapse; TBI, total body irradiation.

^a^ Age, sex and cytogenetics group were forced into the model as adjustment variables regardless of statistical significance.

### Key risk factors in patients undergoing HSCT2

A longer time between HSCT1 and relapse (>6 months) was a favourable prognostic factor for LFS (HR: 0.60 [95% CI: 0.43–0.84], *p* = 0.003), OS (HR: 0.64 [95% CI: 0.44–0.93], *p* = 0.018), RI (HR: 0.59 [95% CI: 0.40–0.87], *p* = 0.008) and GRFS (HR: 0.60 [95% CI: 0.43–0.84], *p* = 0.003). Active disease at HSCT2 significantly impaired LFS (HR: 1.75 [95% CI: 1.23–2.50], *p* = 0.002), OS (HR: 1.72 [95% CI: 1.17–2.52], *p* = 0.005), RI (HR: 1.87 [95% CI: 1.24–2.81], *p* = 0.003) and GRFS (HR: 1.75 [95% CI: 1.23–2.50], *p* = 0.002). MMRD was a significant risk factor for lower OS (HR: 1.65 [95% C:I 1.01–2.69], *p* = 0.044). No impact of the MAC regimen was found for LFS, NRM, RI or GRFS (Table 3; Table S2). These results were further supported by the fact that no significant interaction term between period and identified risk factors has been found, suggesting a consistent effect of time from HSCT1 to relapse, active disease at HSCT2 and MMRD across the two time periods. This also reflects that the effect of the period is not driven by changes in the effects or distribution of the identified risk factors.

### Impact of conditioning regimen on HSCT2 clinical outcomes

Myeloablative TBI was used as part of the conditioning regimen in 34.4% of patients (*n* = 110), with equal repartition between the two periods. In our primary multivariable analysis, TBI showed no significant impact on LFS, OS, NRM, RI or GRFS. We also assessed the effect of treosulfan compared to busulfan on transplant outcomes for patients who underwent HSCT2, focusing on those treated since the increased adoption of treosulfan in clinical practice around 2010. From this time, 70 patients had received treosulfan as part of their conditioning regimen for HSCT2; our multivariable analysis showed no significant difference in key relapse‐ and survival‐related outcomes between treosulfan and busulfan (Table 3; Table S3).

## DISCUSSION

As of now, there is no established treatment plan for post‐transplant relapse of paediatric AML. A second transplantation (HSCT2) remains the only effective chance of cure for most patients in this situation. However, there are significant risks associated with HSCT2, especially a very high relapse rate, alongside significant NRM, which exceeds 20%.^14^, ^22^, ^23^, ^24^ It therefore seems crucial to determine which of these patients would potentially benefit from a second transplant. While large published cohorts of adults provide some insights, their applicability in paediatric settings is limited due to differences in transplantation procedures, AML biology and patient characteristics compared to the adult population.^30^, ^31^

This is the largest retrospective analysis focusing on HSCT2 for paediatric relapsed AML after HSCT1 to date. At 3 years, the LFS and OS for the entire population were 30.2% (95% CI: 24.9–35.5) and 37.5% (95% CI: 31.8–43.2) respectively. Our results revealed better outcomes than previous studies, which reported long‐term LFS and OS rates of 17%–22% and 24%–31% respectively.^21^, ^22^, ^23^, ^24^ Additionally, we observed significant improvement in overall and LFS over time. However, both relapse incidence and NRM rate remain high. As highlighted previously, we found that the two main favourable prognostic factors were both a time between HSCT1 and relapse greater than 6 months and the achievement of a new CR before the second transplant.^22^, ^24^, ^30^, ^31^

According to our data, relapse incidence remains the main issue after a second transplant. In our population, the vast majority of these relapses occurred during the first year post–second transplant, underlying the need to improve the post‐transplant relapse prevention strategies and the selection of candidates for this second allograft. However, it is interesting to note a significant decrease in the incidence of relapse over time, which is probably the main factor in improving LFS and OS during the latest period of our analysis, while NRM incidence remained stable over time despite the increased use of myeloablative conditioning regimens and alternative donors. This likely reflects improved supportive care and prophylactic measures post‐transplant.

Relapse incidence improvement could have several potential explanations: more effective pretransplant strategies enabling deeper pretransplant remissions, more effective conditioning regimens, and finally, more effective prophylactic or pre‐emptive post‐transplant strategies.

Focusing on the conditioning regimen used, we did not find an association between the intensity of conditioning and HSCT2 outcomes. This is similar to previous studies but is also a significant difference from adult cohorts.^21^, ^22^, ^23^, ^30^, ^32^ It is interesting to highlight that among myeloablative conditioning, no modality (TBI, busulfan or treosulfan) has been identified as having a more significant impact than the others on OS, LFS, RI and NRM. Different authors have identified treosulfan as less toxic than TBI and busulfan.^27^, ^33^ However, our analysis was not able to include morbidity data, particularly in terms of veno‐occlusive disease or secondary cancer. Finally, post‐transplant immunomodulation strategies and other strategies aimed at preventing relapses could not be included in our analysis because these data are poorly reported in the registry.

Consistent with previous studies, we did not find any impact of age, gender and donor source on survival.^21^, ^22^, ^23^, ^24^ Regarding selecting the same donor versus a different donor, the data were unavailable for 79 patients, so we did not include the variable in the multivariable analysis. We encountered the same issue with many missing data for Lansky performance status, CMV serostatus (donor and recipient) and in vivo T‐cell depletion. Although these variables appeared relevant in some paediatric and adult studies, we could not include them in our model.^30^, ^34^

To conclude, our data confirm that some paediatric AML patients who relapsed after a first allogeneic transplantation may have prolonged survival after a second transplantation but still face a high risk of relapse. We highlighted significant improvements over time, which could be a starting point for further studies investigating predictive factors of HSCT2. We hypothesise that the increased use of more robust and new strategies (gemtuzumab ozogamicin, venetoclax, hypomethylating agent, maintenance therapy, donor lymphocytes infusion) in the last decade led to better control of disease before HSCT2 and might explain the greater results observed in the second period of our study. However, we cannot prove this since the EBMT registry does not mention the procedure to manage post‐HSCT1 relapses. Another possibility is that earlier detection of relapse through MRD monitoring could result in a low AML disease burden. This supports the need for future collaborative work to collect such data to allow progress in post‐transplant paediatric AML relapses.

## AUTHOR CONTRIBUTIONS

Victor Michel and Nimrod Buchbinder designed the study and drafted the manuscript, with assistance from Pascale Schneider. Mouad Abouqateb analysed the data. Nimrod Buchbinder, Franco Locatelli, Alexei Maschan, Robert Wynn, Franca Fagioli, Marco Zecca, Charlotte Jubert, Birgitta Versluys, Petr Sedlacek, Marta Gonzalez Vicent, Alessandra Biffi, Gérard Michel, Oana Mirci‐Danicar, Wolfgang Holter and Krzysztof Kałwak contributed to the registration. Krzysztof Kałwak, Katharina Kleinschmidt, Mouad Abouqateb, Jacques‐Emmanuel Galimard, Mouad Abouqateb and Arnaud Dalissier are responsible for data management. All authors discussed and approved the manuscript.

## FUNDING INFORMATION

This research received no specific grant from any funding agency in the public, commercial, or not‐for‐profit sectors.

## CONFLICT OF INTEREST STATEMENT

Alexei Maschan: Lecturer's honoraria from AstraZeneca and Generium; Meeting attendance support from AstraZeneca. Mouad Abouqateb: Lecturer's Honoraria for from Jazz Pharmaceutical (JP) and Vertex; Meeting attendance support from JP and Novo Nordisk. Charlotte Jubert: Lecturer's Honoraria from Jazz Pharmaceutical; Meeting attendance support from Pierre Fabre, Jazz Pharmaceutical France, Sanofi Aventis France, Servier. Katharina Kleinschmidt: Honoraria for speaker's bureau involvement and meeting attendance support from Medac. Consulting fees from Sobi and Vertex. Writing support honouraria from Jazz Pharmaceutical. Meeting attendance support from Medac and Sobi. Marta Gonzalez Vicent: Lecturer's Honoraria and meeting attendance support from Sobi and Jazz Pharmaceutical. Nimrod Buchbinder: Meeting attendance support from Medac, Servier and Jazz Pharmaceutical; Educational event speaker honoraria from Servier.

## ETHICS STATEMENT

This study was approved by the institutional review board (IRB) of the Paediatric Disease Working Party of the European Bone Marrow Transplantation Society.

## PATIENT CONSENT STATEMENT

All patients and/or guardians provided written informed consent for data entry into the ProMISe database and its use for analysis, per the Declaration of Helsinki.

## CLINICAL TRIAL REGISTRATION (INCLUDING TRIAL NUMBER)

The study number (EBMT NUMBER) is 8417050.

## Supporting information


Data S1.

## Data Availability

Data cannot be shared unless a specific request is sent to the EBMT.
