# Supplementary material for: Outcomes after a second allogeneic haematopoietic stem cell transplant for relapsed paediatric acute myeloid leukaemia improved over time: A study from the EBMT Paediatric Diseases Working Party
Source: Br J Haematol. 2025 Sep 30;207(6):2496–506. doi: 10.1111/bjh.70167 (PMC12710151; doi:10.1111/bjh.70167)
Supplement: Supplementary file 1 — Data S1. [file BJH-207-2496-s001.docx]

**Appendix**

**Supplemental Table 1.** Additional details on changes in patient and second transplant characteristics over time

|  | [2000-2013] (N=168) | [2014-2022] (N=177) | Total (N=345) | p-value |
| --- | --- | --- | --- | --- |
| **Donor Sex** |  |  |  | 0.69 |
| Female | 66 (42.9%) | 51 (40.5%) | 117 (41.8%) |  |
| Male | 88 (57.1%) | 75 (59.5%) | 163 (58.2%) |  |
| Not applicable In CB | 10 | 46 | 56 |  |
| Missing count | 4 | 5 | 9 |  |
| **Cell Source** |  |  |  | < 0.01 |
| BM | 59 (35.3%) | 53 (29.9%) | 112 (32.6%) |  |
| CB | 11 (6.6%) | 46 (26.0%) | 57 (16.6%) |  |
| PB | 97 (58.1%) | 78 (44.1%) | 175 (50.9%) |  |
| Missing count | 1 | 0 | 1 |  |
| **In vivo T cell depletion** |  |  |  | 0.11 |
| ATG | 56 (44.1%) | 84 (52.5%) | 140 (48.8%) |  |
| Campath | 6 (4.7%) | 2 (1.2%) | 8 (2.8%) |  |
| No | 65 (51.2%) | 74 (46.2%) | 139 (48.4%) |  |
| Missing count | 41 | 17 | 58 |  |
| **Ex-vivo T-cell depletion** |  |  |  | 0.04 |
| No | 141 (88.1%) | 138 (79.8%) | 279 (83.8%) |  |
| Yes | 19 (11.9%) | 35 (20.2%) | 54 (16.2%) |  |
| Missing count | 8 | 4 | 12 |  |
| **Conditioning Regimen Group** |  |  |  | < 0.01 |
| TBI based | 58 (38.9%) | 52 (30.4%) | 110 (34.4%) |  |
| Bu based | 29 (19.5%) | 45 (26.3%) | 74 (23.1%) |  |
| Treo based | 23 (15.4%) | 53 (31.0%) | 76 (23.8%) |  |
| Other (Flu, Mel, Thio) | 39 (26.2%) | 21 (12.3%) | 60 (18.8%) |  |
| Missing count | 19 | 6 | 25 |  |
| **GVHD Prevention Regimen** |  |  |  |  |
| ATG only | 11 (8.7%) | 19 (11.9%) | 30 (10.5%) |  |
| ATG+CSA based | 11 (8.7%) | 16 (10.0%) | 27 (9.4%) |  |
| ATG+CSA+MMF based | 7 (5.5%) | 12 (7.5%) | 19 (6.6%) |  |
| ATG+CSA+MTX based | 22 (17.3%) | 30 (18.8%) | 52 (18.1%) |  |
| ATG+MMF based | 3 (2.4%) | 6 (3.8%) | 9 (3.1%) |  |
| ATG+MTX based | 2 (1.6%) | 1 (0.6%) | 3 (1.0%) |  |
| CSA based | 40 (31.5%) | 10 (6.2%) | 50 (17.4%) |  |
| CSA+MMF based | 10 (7.9%) | 32 (20.0%) | 42 (14.6%) |  |
| CSA+MTX based | 14 (11.0%) | 15 (9.4%) | 29 (10.1%) |  |
| MMF based | 6 (4.7%) | 7 (4.4%) | 13 (4.5%) |  |
| MTX based | 0 (0.0%) | 1 (0.6%) | 1 (0.3%) |  |
| Other | 1 (0.8%) | 2 (1.2%) | 3 (1.0%) |  |
| TACRO/SIRO based | 0 (0.0%) | 9 (5.6%) | 9 (3.1%) |  |
| Missing count | 41 | 17 | 58 |  |
| **Conditioning Regimen** |  |  |  |  |
| Bu+Other | 1 (0.7%) | 1 (0.6%) | 2 (0.6%) |  |
| BuCy based | 9 (6.0%) | 9 (5.3%) | 18 (5.6%) |  |
| BuFlu based | 11 (7.4%) | 14 (8.2%) | 25 (7.8%) |  |
| BuFluThio based | 3 (2.0%) | 12 (7.0%) | 15 (4.7%) |  |
| BuMel based | 5 (3.4%) | 9 (5.3%) | 14 (4.4%) |  |
| Flu or Cy based | 9 (6.0%) | 2 (1.2%) | 11 (3.4%) |  |
| FluMel based | 15 (10.1%) | 9 (5.3%) | 24 (7.5%) |  |
| FluMel+Thio based | 5 (3.4%) | 3 (1.8%) | 8 (2.5%) |  |
| Mel/Thio based | 5 (3.4%) | 4 (2.3%) | 9 (2.8%) |  |
| MelThio based | 5 (3.4%) | 3 (1.8%) | 8 (2.5%) |  |
| TBI based | 46 (30.9%) | 32 (18.7%) | 78 (24.4%) |  |
| TBI+Thio/Mel based | 11 (7.4%) | 18 (10.5%) | 29 (9.1%) |  |
| TBI+Treo/Bu based | 1 (0.7%) | 2 (1.2%) | 3 (0.9%) |  |
| Treo+other | 2 (1.3%) | 2 (1.2%) | 4 (1.2%) |  |
| TreoFlu based | 6 (4.0%) | 4 (2.3%) | 10 (3.1%) |  |
| TreoFluThio based | 11 (7.4%) | 39 (22.8%) | 50 (15.6%) |  |
| TreoMel/Thio based | 4 (2.7%) | 8 (4.7%) | 12 (3.8%) |  |
| Missing count | 19 | 6 | 25 |  |
| **Center Country** |  |  |  |  |
| Italy | 33 (19.6%) | 33 (18.6%) | 66 (19.1%) |  |
| France | 32 (19.0%) | 22 (12.4%) | 54 (15.7%) |  |
| United Kingdom | 17 (10.1%) | 24 (13.6%) | 41 (11.9%) |  |
| Russia | 10 (6.0%) | 17 (9.6%) | 27 (7.8%) |  |
| Spain | 10 (6.0%) | 9 (5.1%) | 19 (5.5%) |  |
| Poland | 10 (6.0%) | 7 (4.0%) | 17 (4.9%) |  |
| Israel | 9 (5.4%) | 4 (2.3%) | 13 (3.8%) |  |
| Germany | 13 (7.7%) | 2 (1.1%) | 15 (4.3%) |  |
| Saudi Arabia | 0 (0.0%) | 11 (6.2%) | 11 (3.2%) |  |
| Netherlands, The | 1 (0.6%) | 9 (5.1%) | 10 (2.9%) |  |
| Austria | 4 (2.4%) | 5 (2.8%) | 9 (2.6%) |  |
| Turkey | 2 (1.2%) | 8 (4.5%) | 10 (2.9%) |  |
| Belgium | 2 (1.2%) | 4 (2.3%) | 6 (1.7%) |  |
| Czech Republic | 6 (3.6%) | 2 (1.1%) | 8 (2.3%) |  |
| Australia | 0 (0.0%) | 6 (3.4%) | 6 (1.7%) |  |
| Sweden | 3 (1.8%) | 3 (1.7%) | 6 (1.7%) |  |
| Switzerland | 2 (1.2%) | 1 (0.6%) | 3 (0.9%) |  |
| Denmark | 2 (1.2%) | 2 (1.1%) | 4 (1.2%) |  |
| Norway | 2 (1.2%) | 2 (1.1%) | 4 (1.2%) |  |
| Other | 10 (6.0%) | 6 (3.4%) | 16 (4.6%) |  |

PB peripheral blood, BM bone marrow, ATG anti-thymocyte globulin, TBI total body irradiaation, Bu busulfan, Treo treosulfan, Flu fludarabine, Mel melphalan, Thio thiotepa, GVHD: Graft-versus-host disease, CSA: ciclosporin A, MMF: mycophenolate mofetil, MTX: methotrexate, TACRO/SIRO tacrolimus/sirolimus Cy cyclophosphamide

**Supplemental Table 2. .** Multivariable analysis of risk factors for GVH outcomes after second allogeneic stem cell transplantation

| **Characteristic** | **CGVH** | | **aGVH-II/IV** | | **aGVH-III/IV** | |
| --- | --- | --- | --- | --- | --- | --- |
|  | **HR** **(95% CI)***^1^* | **p-value***^2^* | **HR** **(95% CI)***^1^* | **p-value***^2^* | **HR** **(95% CI)***^1^* | **p-value***^2^* |
| **HSCT2 time period** |  |  |  |  |  |  |
| [2000-2013] | — |  | — |  | — |  |
| [2014-2022] | 0.88 (0.43 to 1.80) | 0.73 | 0.89 (0.57 to 1.41) | 0.63 | 0.92 (0.46 to 1.84) | 0.81 |
| **Age at Transplant** | 1.02 (0.95 to 1.09) | 0.57 | 0.99 (0.95 to 1.03) | 0.53 | 0.99 (0.93 to 1.06) | 0.76 |
| **Gender** |  |  |  |  |  |  |
| Male | — |  | — |  | — |  |
| Female | 1.38 (0.72 to 2.64) | 0.33 | 1.19 (0.78 to 1.80) | 0.42 | 1.82 (0.96 to 3.45) | 0.064 |
| **Delay HSCT1-Relapse, months** |  |  |  |  |  |  |
| 0 to 6 | — |  | — |  | — |  |
| More than 6 | 0.74 (0.32 to 1.72) | 0.49 | 0.80 (0.47 to 1.35) | 0.41 | 0.84 (0.37 to 1.91) | 0.68 |
| **Donor Type** |  |  |  |  |  |  |
| Matched Related Donor | — |  | — |  | — |  |
| Mismatched Related Donor | 1.23 (0.41 to 3.67) | 0.71 | 1.66 (0.84 to 3.31) | 0.15 | 1.03 (0.37 to 2.89) | 0.95 |
| Unrelated_Donor | 2.13 (0.88 to 5.14) | 0.094 | 1.67 (0.91 to 3.05) | 0.10 | 1.30 (0.56 to 3.03) | 0.55 |
| **Cytogenetic Risk** |  |  |  |  |  |  |
| Favorable & Intermediate | — |  | — |  | — |  |
| Adverse | 0.42 (0.19 to 0.94) | **0.035*** | 1.47 (0.90 to 2.40) | 0.12 | 0.81 (0.37 to 1.79) | 0.61 |
| Missing | 0.77 (0.34 to 1.78) | 0.55 | 1.17 (0.68 to 2.01) | 0.58 | 0.91 (0.41 to 2.04) | 0.82 |
| **Disease Status at Transplant** |  |  |  |  |  |  |
| CR | — |  | — |  | — |  |
| Active Disease | 0.70 (0.24 to 2.04) | 0.51 | 0.77 (0.44 to 1.35) | 0.36 | 0.74 (0.30 to 1.83) | 0.52 |
| **TBI Regimen** |  |  |  |  |  |  |
| No | — |  | — |  | — |  |
| Yes | 1.20 (0.60 to 2.38) | 0.61 | 1.09 (0.70 to 1.72) | 0.70 | 0.96 (0.48 to 1.92) | 0.91 |
| **MAC Regimen** |  |  |  |  |  |  |
| No | — |  | — |  | — |  |
| Yes | 0.57 (0.26 to 1.22) | 0.15 | 0.68 (0.42 to 1.08) | 0.10 | 0.58 (0.29 to 1.17) | 0.13 |
| *^1^* HR = Hazard Ratio, CI = Confidence Interval | | | | | | |
| *^2^* *p<0.05; **p<0.01; ***p<0.001 | | | | | | |

HR hazard ratio, CI confidence interval, HSCT allogeneic stem cell transplantation, HSCT1 first SCT, HSCT2 second SCT, MRD matched related donor, MMRD mismatched related donor, CR complete remission, Rel relapse, TBI total body irradiation, MAC myeloablative conditioning regimen, Significant values are in bold.

**Supplemental Table 3.** Multivariable analysis Treosulfan vs. Busulfant in second allogeneic stem cell transplant.

| **Characteristic** | **Leukemia Free Survival LFS** | | **Overall Survival OS** | | **Relapse Incidence RI** | | **GRFS** | |
| --- | --- | --- | --- | --- | --- | --- | --- | --- |
|  | **HR** **(95% CI)***^1^* | **p-value***^2^* | **HR** **(95% CI)***^1^* | **p-value***^2^* | **HR** **(95% CI)***^1^* | **p-value***^2^* | **HR** **(95% CI)***^1^* | **p-value***^2^* |
| **Conditioning Regimen** |  |  |  |  |  |  |  |  |
| Busulfan based | — |  | — |  | — |  | — |  |
| Treosulfan based | 0.89 (0.56 to 1.43) | 0.64 | 0.81 (0.49 to 1.34) | 0.41 | 0.82 (0.47 to 1.42) | 0.47 | 0.89 (0.56 to 1.43) | 0.64 |
| **Age at Transplant** | 1.05 (1.00 to 1.10) | **0.047*** | 1.03 (0.98 to 1.08) | 0.25 | 1.06 (1.00 to 1.12) | 0.072 | 1.05 (1.00 to 1.10) | **0.047*** |
| **Delay HSCT1-relapse, months** |  |  |  |  |  |  |  |  |
| 0 to 6 | — |  | — |  | — |  | — |  |
| More than 6 | 0.31 (0.16 to 0.57) | **<0.001***** | 0.38 (0.19 to 0.75) | **0.005**** | 0.31 (0.15 to 0.65) | **0.002**** | 0.31 (0.16 to 0.57) | **<0.001***** |
| **Year of Transplant** | 0.92 (0.86 to 0.98) | **0.014*** | 0.93 (0.86 to 1.00) | **0.038*** | 0.93 (0.86 to 1.01) | 0.075 | 0.92 (0.86 to 0.98) | **0.014*** |
| **Disease Status at Transplant** |  |  |  |  |  |  |  |  |
| CR | — |  | — |  | — |  | — |  |
| Active Disease | 1.52 (0.84 to 2.74) | 0.17 | 1.16 (0.60 to 2.25) | 0.65 | 2.06 (1.06 to 4.00) | **0.034*** | 1.52 (0.84 to 2.74) | 0.17 |
| *^1^* HR = Hazard Ratio, CI = Confidence Interval | | | | | | | | |
| *^2^* *p<0.05; **p<0.01; ***p<0.001 | | | | | | | | |

HR hazard ratio, CI confidence interval, HSCT allogeneic stem cell transplantation, HSCT1 first SCT, HSCT2 second SCT, CR complete remission, Rel relapse. Significant values are in bold.
